# TGFβ/Smad3 regulates proliferation and apoptosis through IRS-1 inhibition in colon cancer cells

**DOI:** 10.1371/journal.pone.0176096

**Published:** 2017-04-17

**Authors:** Katie L. Bailey, Ekta Agarwal, Sanjib Chowdhury, Jiangtao Luo, Michael G. Brattain, Jennifer D. Black, Jing Wang

**Affiliations:** 1 Eppley Institute for Research in Cancer and Allied Diseases, University of Nebraska Medical Center, Omaha, Nebraska, United States of America; 2 Department of Biochemistry and Molecular Biology, University of Nebraska Medical Center, Nebraska, United States of America; 3 Wistar Institute, Philadelphia, Pennsylvania; 4 Section of Gastroenterology, Department of Medicine, Boston University Medical Center, Boston, Massachusetts, United States of America; 5 Department of Biostatistics, College of Public Health, University of Nebraska Medical Center, Omaha, Nebraska, United States of America; 6 Department of Genetics, Cell Biology and Anatomy, University of Nebraska Medical Center, Omaha, Nebraska, United States of America; University of South Alabama Mitchell Cancer Institute, UNITED STATES

## Abstract

In this study, we have uncovered a novel crosstalk between TGFβ and IGF-1R signaling pathways. We show for the first time that expression and activation of IRS-1, an IGF-1R adaptor protein, is decreased by TGFβ/Smad3 signaling. Loss or attenuation of TGFβ activation leads to elevated expression and phosphorylation of IRS-1 in colon cancer cells, resulting in enhanced cell proliferation, decreased apoptosis and increased tumor growth *in vitro* and *in vivo*. Downregulation of IRS-1 expression reversed Smad3 knockdown-mediated oncogenic phenotypes, indicating that TGFβ/Smad3 signaling inhibits cell proliferation and increases apoptosis at least partially through the inhibition of IRS-1 expression and activation. Additionally, the TGFβ/Smad3/IRS-1 signaling axis regulates expression of cyclin D1 and XIAP, which may contribute to TGFβ/Smad3/IRS-1-mediated cell cycle progression and survival. Given that loss of TGFβ signaling occurs frequently in colon cancer, an important implication of our study is that IRS-1 could be a potential therapeutic target for colon cancer treatment.

## Introduction

Transforming growth factor β (TGFβ) signaling regulates many important cellular functions, including differentiation, proliferation, migration and apoptosis [1]. The TGFβ pathway is activated upon TGFβ ligand binding to TGFβ receptor type II (RII), which phosphorylates and activates TGFβ receptor type I (RI), leading to phosphorylation of Smad2/3 (R-Smads). R-Smads then complex with Smad4 and translocate to the nucleus to regulate expression of various target genes [2].

TGFβ signaling has been shown to inhibit cell proliferation, induce apoptosis and suppress tumorigenicity in many colon cancer cell lines [3]. In addition, abrogation of TGFβ signaling increases metastasis, whereas enhanced TGFβ signaling suppresses metastasis in an orthotopic model of colon cancer [1,4]. In genetic mouse models, Smad3 or Smad4 mutation increases malignancy and invasiveness of intestinal tumors in APC ^min/+^ or APC ^Δ716/+^ mice [5,6], pointing to a significant role of TGFβ signaling in repressing malignant progression in colon tumors. Indeed, TGFβ signaling is defective in 30%–40% of colon cancer patients due to defects in TGFβ RII or Smads [7,8,9], and loss or reduction of TGFβ signaling is tightly associated with the development of metastasis in patient sample studies [10,11].

In addition to inactivation of TGFβ signaling, cancer cells evade tumor suppressive TGFβ signaling by activation of oncogenic pathways [1,12]. An oncogenic pathway that is activated in 30% of human colon cancer is the type I Insulin Like Growth Factor Receptor (IGF-1R) signaling pathway [12,13]. IGF-1R signaling is activated through the binding of Insulin Like Growth Factor (IGF) to the receptor, leading to receptor autophosphorylation and binding of the adaptor proteins, Insulin Receptor Substrates (IRS)-1 and 2 [12]. IRS-1 and IRS-2 have been shown to play differential roles in promoting tumorigenesis [12,14,15]. Specifically, IRS-1 is constitutively activated in a number of solid tumors including breast, colon, hepatocellular and lung cancers [16,17,18]. Overexpression of IRS-1 leads to increased proliferation, mitogenesis, migration and anti-apoptotic signaling [19,20,21,22,23]. In mouse mammary gland, overexpression of IRS-1 leads to mammary hyperplasia and tumorigenesis [18]. On the other hand, knockdown of IRS-1 leads to reduced cell migration and decreased tumor growth [16,20].

One of the main downstream signaling pathways activated by phosphorylated IRS-1 is the phosphoinositide 3-kinase (PI3K)/AKT pathway [12,24]. When IRS-1 is overexpressed, the PI3K/AKT pathway becomes constitutively activated, leading to increased cell survival and tumor growth [22]. It has been shown that PI3K/AKT signaling upregulates expression of x-linked inhibitor of apoptosis protein (XIAP) and inhibits cell death [1,3]. XIAP inhibits apoptosis through direct interaction with caspases and is overexpressed in various types of cancer including colon, prostate, cervical and hepatocellular cancer, leading to chemotherapy resistance in many patients [25,26,27].

In addition to activation of PI3K/AKT signaling, IRS-1 also translocates to the nucleus, where it acts as a transcriptional co-factor and binds to the cyclin D1 promoter to increase its transcription [19]. Cyclin D1 is an important regulator of cell cycle progression, which inactivates the retinoblastoma protein to promote cell entry into G1 phase [28]. Overexpression of cyclin D1 is one of the most common abnormalities in human cancer [28]. In colon cancer, overexpression of cyclin D1 is observed in 55% of patients [29]. Cyclin D1 overexpression leads to increased proliferation, angiogenesis and cell survival [29], which are essential to maintain the malignant phenotype in colon cancer [30].

It has been previously reported that TGFβ induces phosphorylation of IRS-2 in Mv1Lu cells and that transfection with IRS-1 or IRS-2 cDNA confers sensitivity to growth inhibition by TGFβ, suggesting that IRS proteins are involved in TGFβ-mediated growth inhibition [22,31]. Other studies have shown that IRS-1 prevents TGFβ-induced apoptosis [22]. Thus, understanding of the crosstalk between TGFβ and IGF-1R/IRS-1 signaling remains limited. In this study, we show that abrogation of TGFβ signaling increased expression and phosphorylation of IRS-1 in colon cancer cells *in vitro* and in a tumor xenograft model *in vivo*. Further studies indicate that TGFβ/Smad3 suppresses expression and phosphorylation of IRS-1. Knockdown of IRS-1 expression in Smad3 knockdown cells decreased cell proliferation and increased apoptosis under stress, reversing Smad3 knockdown-mediated oncogenic phenotypes. The underlying mechanism involves regulation of XIAP and cyclinD1 expression by the TGFβ/Smad3/IRS-1 signaling axis. Our studies identify a novel crosstalk between TGFβ and IGF-1R signaling through the adaptor protein, IRS-1, indicating that one of the mechanisms by which TGFβ elicits its tumor suppressor function is through inhibition of IRS-1 expression and activation in colon cancer cells. Therefore, IRS-1 could be a potential therapeutic target for colon cancer treatment.

## Results

### Abrogation of TGFβ signaling leads to increased expression and activation of IRS-1 in colon cancer cells *in vitro* and *in vivo*

**Fig 1:**
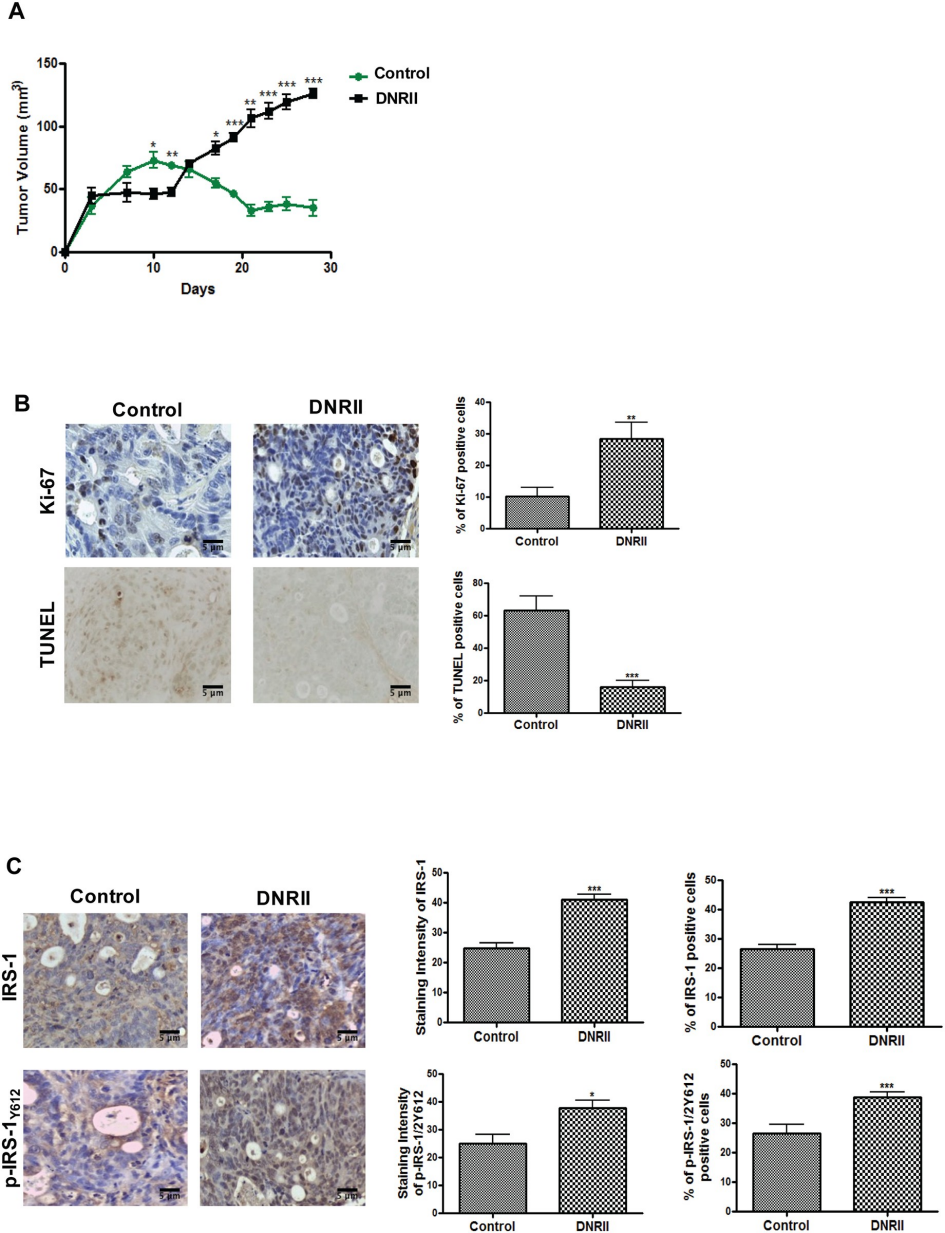
Abrogation of TGFβ signaling led to increased expression and activation of IRS-1 in colon cancer cells *in vivo*. (A) Xenograft tumor growth curves of FET control and DNRII cells are shown. n = 10. **(**B) Representative images of Ki67 and TUNEL staining are shown (left). Percentage of positive staining cells was determined (right). (C) Representative images of IRS-1 and pIRS1Y612 staining in xenograft tumors are shown (left). Quantification of staining intensity and percentage of positive cells was performed (right). Scale bars, 5 μm. The data are presented as the mean ± SD. *P < 0.05, ** *P* < 0.01, *** *P* < 0.001.

As reported previously, FET colon cancer cells, isolated from a well-differentiated early stage colon tumor, express autocrine TGFβ signaling and are sensitive to TGFβ-mediated growth inhibition and apoptosis [32]. When injected subcutaneously into athymic nude mice in a xenograft model *in vivo*, FET cells formed a nodule first, which then regressed by day 12. However, abrogation of TGFβ signaling in FET cells by expression of dominant negative RII (DNRII) led to sustained tumor growth (Fig 1A) [32]. Thus, TGFβ signaling appears to suppress tumorigenesis in colon cancer cells. Ki67 and TUNEL staining were performed to determine the proliferative and apoptotic indices within tumors respectively, which showed an increased number of proliferative cells and fewer apoptotic cells in DNRII tumors than in control tumors (Fig 1B, left panels). Quantification of percentage of positively stained cells revealed an approximately 3-fold increase in proliferative cells and an almost 75% decrease in apoptotic cells in DNRII tumors compared with control tumors (Fig 1B, right panels). These results indicate that the promoting effect of inactivation of TGFβ signaling on tumor growth is a combined result of increased proliferation and decreased apoptosis.

It has been shown that IGF-1R signaling is important for proliferation of colon cancer cells and that IRS-1 is one of the main adaptor proteins required for IGF-1R signaling [33,34]. To determine the mechanisms underlying the suppressive effect of TGFβ signaling on growth of colon cancer cells in the xenograft studies, we determined the expression and phosphorylation of IRS-1 in xenograft tumors by immunohistochemistry (IHC) staining. DNRII tumors showed more intense staining for IRS-1 and more IRS-1 positive cells than control tumors (Fig 1C, left panels). Quantification of the data revealed that the average intensity of IRS-1 staining and the percentage of IRS-1 positive cells were approximately 71% and 62% higher in DNRII tumors than in control tumors, respectively (Fig 1C, right panels). Activation of IRS-1, as reflected by its phosphorylation, was also determined by examining the levels of IRS-1 phosphorylated at tyrosine 612 (Y612). Quantification demonstrated that the average intensity of p-IRS-1-Y612 staining and the percentage of p-IRS-1-Y612 positive cells were approximately 48% and 46% higher in DNRII tumors than in control tumors, respectively (Fig 1C). Taken together, these results indicated that abrogation of TGFβ signaling led to increased expression and activation of IRS-1 in colon cancer cells *in vivo*, suggesting that TGFβ may regulate IRS-1 expression and activation.

### TGFβ signaling inhibits expression and phosphorylation of IRS-1 in colon cancer cells

**Fig 2:**
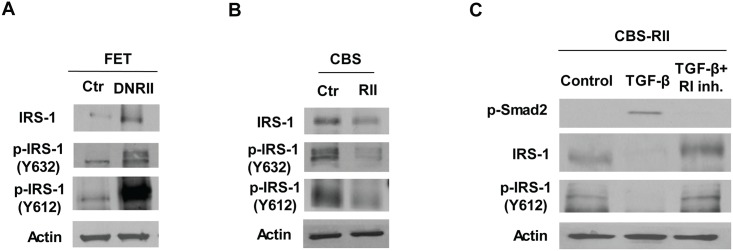
TGFβ inhibits expression and phosphorylation of IRS-1 in colon cancer cells. **(**A) Expression and phosphorylation of IRS-1 at both Y632 and Y612 were higher in FET-DNRII cells than in control cells, as shown by western blot analysis. (B) Expression and phosphorylation of IRS-1 at both Y632 and Y612 were lower in CBS-RII cells than in the control cells, as shown by western blot analysis. (C) TGFβ treatment of CBS-RII cells decreased expression and phosphorylation of IRS-1, an effect that was prevented by the addition of TGFβ RI kinase inhibitor.

To explore the regulation of IRS-1 by TGFβ signaling, expression and phosphorylation of IRS-1 were determined in FET control and DNRII cells. Consistent with *in vivo* findings, IRS-1 expression and phosphorylation were upregulated in DNRII cells compared with control cells (Fig 2A).

As described previously, exogenous expression of TGFβ RII in CBS colon cancer cells (CBS-RII) enhanced TGFβ signaling, resulting in reduced proliferation, increased apoptosis and suppressed metastatic potential in an orthotopic model *in vivo* [4]. We therefore examined expression and activation of IRS-1 in CBS control and CBS-RII cells. The results show that IRS-1 expression and phosphorylation are decreased in CBS-RII cells compared with control cells (Fig 2B), indicating that enhancing TGFβ signaling by TGFβ RII upregulation suppresses the expression and activation of IRS-1 in colon cancer cells.

To directly demonstrate whether TGFβ regulates expression and activation of IRS-1, CBS-RII cells were treated with TGFβ in the presence or absence of a TGFβ RI kinase inhibitor. As expected, phosphorylation of Smad2 was increased upon TGFβ treatment and addition of the TGFβ RI inhibitor prevented TGFβ-induced Smad2 phosphorylation (Fig 2C). In addition, TGFβ treatment decreased the expression and phosphorylation of IRS-1 in CBS-RII cells and the RI inhibitor prevented the inhibitory effect of TGFβ (Fig 2C). These results demonstrate that TGFβ suppresses expression and activation of IRS-1 in colon cancer cells.

### TGFβ/Smad3 elicits its function through the inhibition of IRS-1 expression/activation

**Fig 3:**
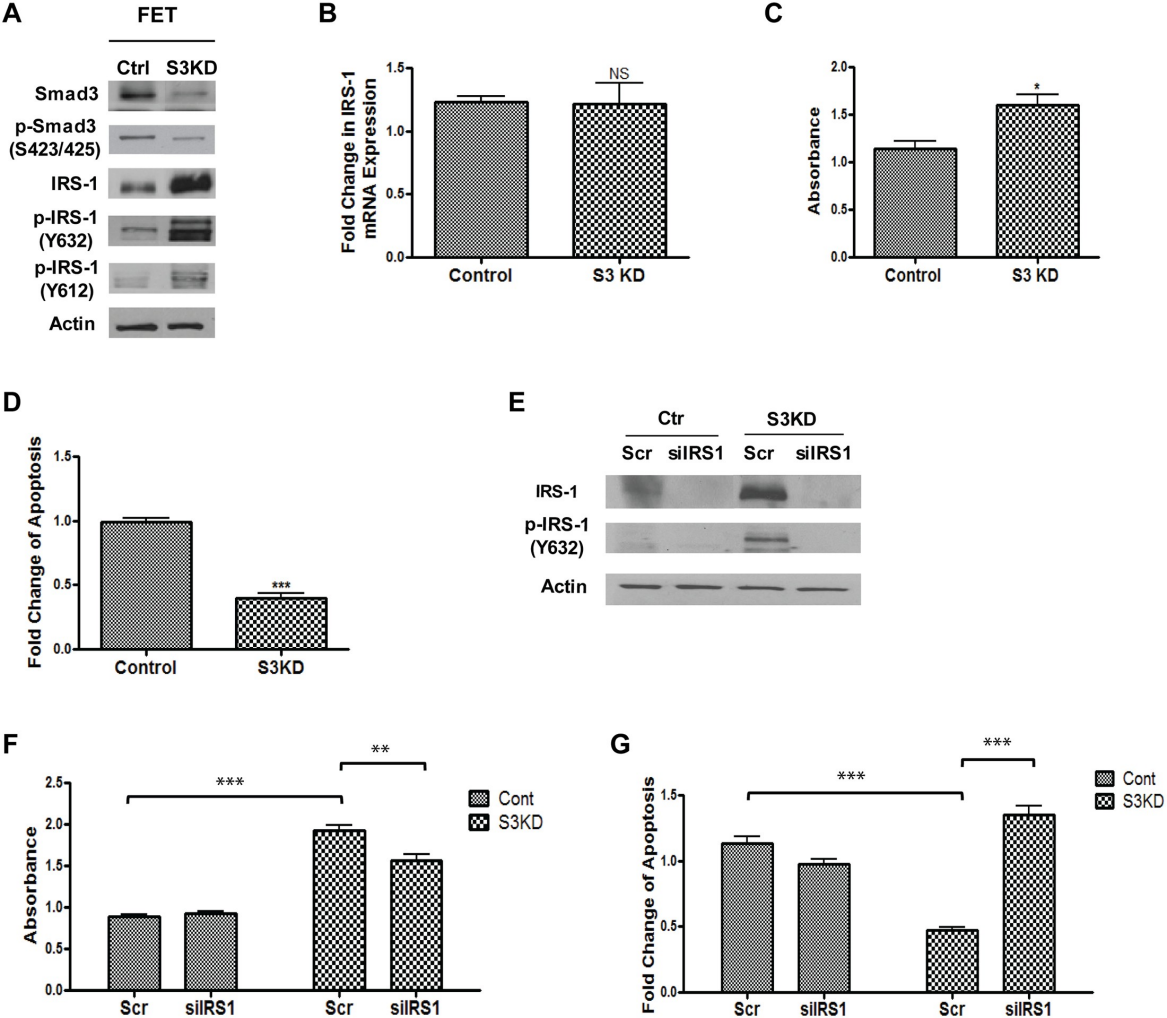
Knockdown of Smad3 led to increased expression and phosphorylation of IRS-1, which contributed to Smad3 knockdown-induced increased proliferation and decreased apoptosis under stress. (A) knockdown of Smad3 in FET cells attenuated TGFβ signaling and increased IRS-1 expression and phosphorylation. (B) knockdown of Smad3 in FET cells had little effect on IRS-1 mRNA expression. (C) MTT assays showed that cell proliferation was increased in Smad3 knockdown cells compared with control cells. (D) DNA fragmentation assays showed protection from apoptosis in Smad3 knockdown cells compared with control cells under GFDS. (E) A siRNA pool targeting IRS-1 was transfected into FET-Smad3 knockdown and control cells. Expression and phosphorylation of IRS-1 were markedly reduced in siRNA-transfected cells compared with scrambled siRNA-transfected control cells. (F) MTT assays showed that knockdown of IRS-1 expression partially prevented Smad3 knockdown-mediated increased proliferation of FET cells. (G) DNA fragmentation assays showed that knockdown of IRS-1 expression abrogated Smad3 knockdown-mediated reduction of apoptosis under GFDS. For F and G, two-way ANOVA was used to compare the difference between Cont Scr and S3KD Scr and Student’s t-test was used to compare the difference between S3KD Scr and siIRS-1. The data are presented as the mean ± SD of three replicates. *P < 0.05, ** *P* < 0.01, *** *P* < 0.001.

Since Smad3 is an important effector in TGFβ signaling, we next determined whether downregulation of Smad3 would affect IRS-1 expression and activation in colon cancer cells. Smad3 was knocked down in FET cells using shRNA (designated FET/S3KD, Fig 3A). A scrambled shRNA was used as a control. As expected, Smad3 knockdown reduced TGFβ signaling as reflected in decreased overall levels of Smad3 phosphorylation (Fig 3A). Notably, expression of IRS-1 was markedly increased in Smad3 KD cells (Fig 3A), accompanied by increased levels of phosphorylation at both Y632 and Y612 (Fig 3A). These results were consistent with those obtained in FET/DNRII cells (Fig 2A). Of note, IRS-1 mRNA expression was not affected by Smad3 knockdown (Fig 3B), suggesting that Smad3 regulates IRS-1 expression primarily at the post-transcriptional level. Together the data indicates that attenuation of TGFβ signaling by inactivation of TGFβ RII or decreasing Smad3 expression results in increased IRS-1 expression and activation.

As expected, knockdown of Smad3 expression also resulted in increased proliferation and decreased apoptosis in the context of growth factors and serum deprivation stress (Fig 3C and 3D). To determine whether IRS-1 plays a role in the pro-proliferative and anti-apoptotic effects of Smad deficiency, expression of IRS-1 was knocked down in FET/S3KD and control cells using a pool of siRNA-targeting IRS-1. Levels of total and phosphorylated IRS-1 were markedly reduced by IRS-1 siRNAs in FET/S3KD and control cells (Fig 3E). Scrambled or IRS-1 siRNA treated FET control and FET/S3KD cells were subjected to growth factor and serum deprivation stress (GFDS) and effects on proliferation and apoptosis were determined. IRS-1 knockdown significantly reduced cell proliferation and increased stress-induced apoptosis in FET/S3KD cells while having little effect in control cells (Fig 3F and 3G). The failure to affect these cellular processes in control cells likely reflects the low basal levels of p-IRS-1 and the involvement of other growth regulatory pathways. Taken together, our studies demonstrate that TGFβ/Smad3 inhibits cell proliferation and induces apoptosis through down-regulation of IRS-1 expression/activation in colon cancer cells.

**Fig 4:**
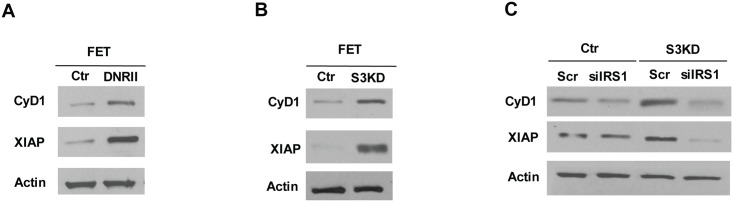
The TGFβ/Smad3/IRS-1 signaling axis regulates expression of cyclin D1 and XIAP. (A&B) Expression of cyclin D1 and XIAP was higher in FET-DNRII (A) and Smad3 knockdown (B) cells. (C) Knockdown of IRS-1 led to reduced expression of cyclin D1 and XIAP in Smad3 knockdown cells.

To determine the mechanism by which the TGFβ/Smad3/IRS-1 axis regulates cell proliferation and survival, expression of important effectors in cell cycle progression and apoptosis were examined. Expression of cyclin D1 and XIAP was markedly increased in DNRII and Smad3 KD cells (Fig 4A and 4B). IRS-1 siRNAs decreased the expression of these molecules in Smad3 KD cells, consistent with effects on cell proliferation and survival, but had little effect in control cells (Fig 4C). These results indicate that TGFβ/Smad3 suppresses cyclin D1 and XIAP expression through inhibition of IRS-1 expression/activation, suggesting that cyclin D1 and XIAP may be involved in the TGFβ/Smad3/IRS-1 signaling axis regulating cell proliferation and survival.

## Discussion

**Fig 5:**
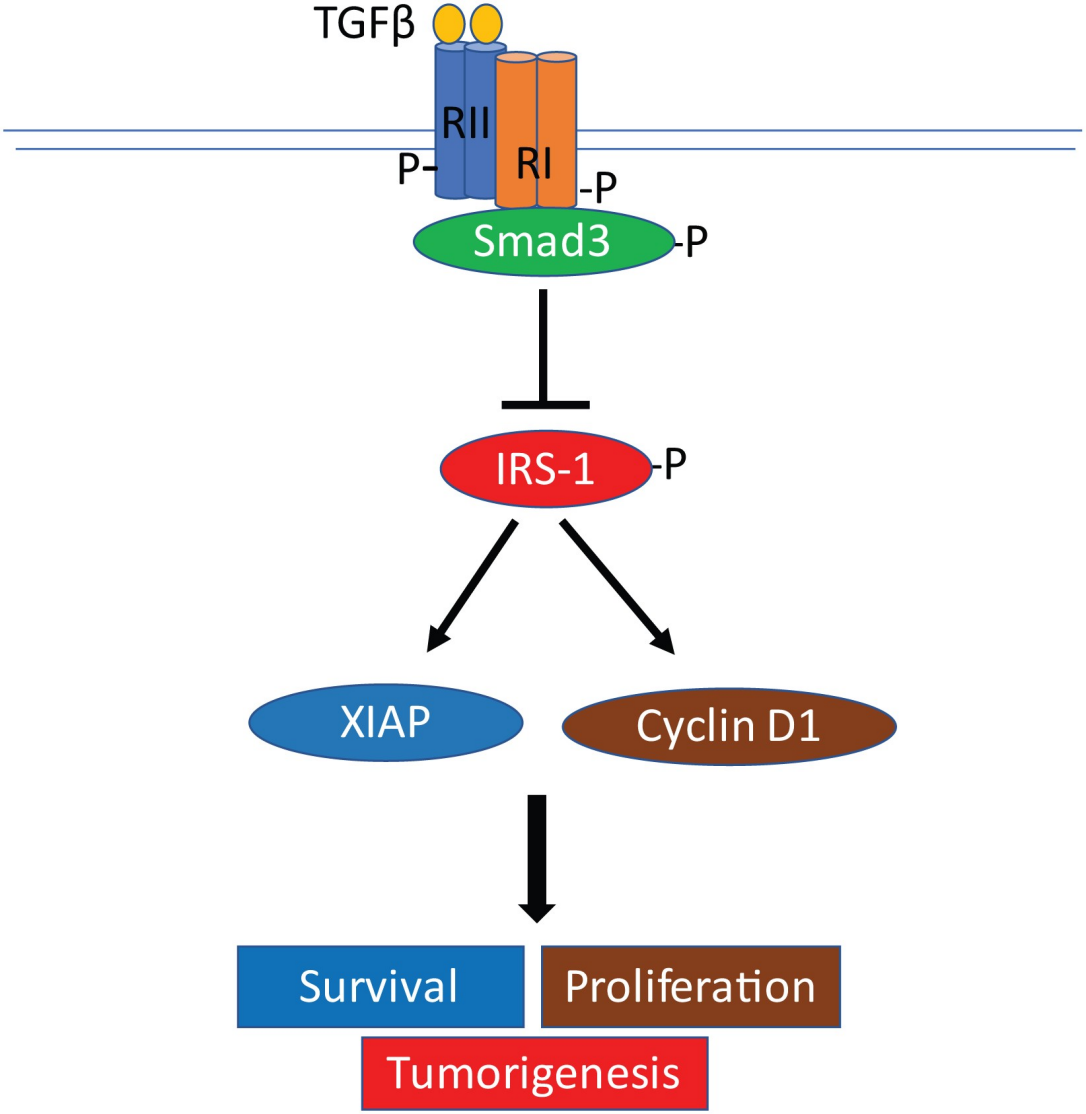
A proposed model of crosstalk between TGFβ signaling and IRS-1 in the regulation of XIAP and cyclin D1 expression, cell survival, proliferation and tumorigenesis in colon cancer cells.

In this study, we have uncovered a novel crosstalk between TGF-β and IGF-1R signaling pathways through the adaptor protein, IRS-1, in colon cancer cells. In a xenograft tumor model, abrogation of TGFβ signaling led to increased colon cancer cell proliferation, decreased apoptosis and enhanced tumor growth *in vivo*, associated with elevated expression and activation of IRS-1. These results indicate that TGFβ signaling regulates IRS-1 expression and activation and that IRS-1 may be a target of TGFβ-mediated tumor suppressor function. Further studies showed that TGFβ signaling suppresses expression and phosphorylation of IRS-1 in colon cancer cells. Knockdown of Smad3 attenuated TGFβ signaling and increased IRS-1 expression and activation, leading to increased cell proliferation and reduced apoptosis under stress. Additional studies indicated that knockdown of IRS-1 expression prevented the effects of Smad3-deficiency on cell proliferation and apoptosis, indicating that TGFβ/Smad3 mediates tumor suppressor function through the inhibition of IRS-1 expression and activation. Mechanistically, the TGFβ/Smad3/IRS-1 axis regulates expression of cyclin D1 and XIAP, which play important roles in cell proliferation and survival, respectively. Taken together, our study suggests a novel model of crosstalk between TGFβ/Smad3 signaling and IRS-1 in colon cancer cells (Fig 5). In this model, TGFβ/Smad3 inhibits IRS-1 expression/activation, which leads to suppression of XIAP and cyclin D1 expression. Consequently, TGFβ signaling elicits its tumor suppressor function through inhibiting survival, proliferation and tumorigenesis. Given that the TGFβ signaling pathway is lost in approximately 30% of colon cancer patients and that IRS-1 has been implicated in the progression of various cancers including colon cancer [16,17,35,36], our study suggests that IRS-1 could be a potential therapeutic target in colon cancer management.

It has been reported that IRS-1 expression is increased in colon adenomas compared with normal colon epithelium [17]. Tumor suppressor microRNA, miR-145, has been shown to downregulate IRS-1 expression in the colon [37,38,39]. In addition, miR-128 suppresses cell growth and metastasis by targeting IRS-1 in colon cancer [40]. Furthermore, miR-126 regulates cell proliferation, migration and invasion of colon cancer cells by targeting IRS-1 [36]. In our study, we show, for the first time, that TGFβ signaling inhibits IRS-1 expression and activation in colon cancer cells *in vitro* and *in vivo* and that upregulated IRS-1 contributes to attenuation of TGFβ signaling-mediated tumor suppressive phenotypes. The frequent loss of TGFβ signaling in colon cancer may account for increased IRS-1 expression. Therefore, our study uncovers another mechanism by which IRS-1 expression is regulated in colon cancer.

Our findings further show that TGFβ/Smad3 inhibits IRS-1 protein expression (Figs 2 and 3A) but has little effect on IRS-1 mRNA levels (Fig 3B), indicating that TGFβ/Smad3 signaling regulates IRS-1 expression at the post-transcriptional level. Since many miRNAs have been shown to regulate IRS-1 expression [36,37,38,39,40], it is possible that TGFβ/Smad3 signaling may mediate expression of those miRNAs. In addition, it has been shown that degradation of IRS-1 protein is regulated by ubiquitination. For example, Cbl-b is an E3 ubiquitin ligase that has been reported to specifically promote the degradation of IRS-1 protein [41]. Further studies are needed to investigate whether TGFβ/Smad3 regulates Cbl-b expression and/or activity in colon cancer cells and whether Cbl-b is responsible for suppression of IRS-1 expression by TGFβ/Smad3 signaling.

Overexpression of IRS-1 has been associated with inhibition of cell death and increased proliferation in different cell types [21,22,42]. One of the main downstream pathways activated by IRS-1 is the PI3K/AKT pathway [12,24]. Additional mediators of IRS-1-mediated function have been reported, including ERK signaling and Sox9 expression [36,43]. Here we show that IRS-1 regulates expression of cyclin D1 and XIAP in colon cancer cells. Given their function in regulating cell cycle and survival, effects on cyclin D1 and XIAP may contribute to IRS-1 mediated enhancement of cell proliferation and inhibition of apoptosis in colon cancer cells.

In summary, we have uncovered a novel crosstalk between TGFβ and IGF-1R signaling. This study shows, for the first time, that expression and phosphorylation of IRS-1, an IGF-1R adaptor protein, is decreased by TGFβ/Smad3 signaling. Loss or attenuation of TGFβ signaling leads to elevated expression and activation of IRS-1, resulting in enhanced cell proliferation, decreased apoptosis and increased tumor growth *in vitro* and *in vivo*. Down-regulation of IRS-1 expression reversed attenuated TGFβ signaling-mediated phenotypes *in vitro*. Since loss of TGFβ signaling is frequent in colon cancer, our study points to IRS-1 as a potential therapeutic target for colon cancer management.

## Materials and methods

### Cell lines and reagents

The human colon cancer cell lines, FET, FET-DNRII, CBS, and CBS-RII, were described previously [32]. All cells were cultured in McCoy’s 5A serum-free medium (Sigma) supplemented with 10 ng/ml epidermal growth factor (EGF), 20 μg/ml insulin, and 4 μg/ml transferrin [44]. When cells were subjected to growth factor deprivation stress (GFDS), they were cultured in McCoy’s 5A serum-free medium in the absence of EGF, insulin and transferrin. Cells were maintained at 37°C in a humidified incubator with 5% CO_2_. TGFβ and the TGFβ RI kinase inhibitor were obtained from R&D Systems and Calbiochem respectively. The following antibodies were used in this study: anti-IRS-1 (1:1000), Cell Signaling #2382, anti-p-IRS-1Y632 (1:600), Santa Cruz sc-17196, anti-p-IRS-1/2Y612 (1:600), Santa Cruz sc-17195-R, anti-Smad3 (1:1000), Cell Signaling #9523, anti-p-Smad3S423/425 (1:1000), Cell Signaling #9520, anti-p-Smad2S465/467 (1:1000), Cell Signaling #3101, anti-XIAP (1:1000), Cell Signaling #14334, anti-Cyclin D1 (1:1000), Cell Signaling #2978 and anti-Actin (1:1000), Sigma #A2066.

### Knockdown of Smad3 expression

FET cells were infected with a retroviral shRNA vector targeting Smad3 as described previously [45]. A scrambled shRNA was used as a control. Smad3 knockdown was confirmed by western blot analysis.

### Transient transfection of siRNAs

IRS-1 ON-TARGETplus Human SMARTpool siRNA IRS-1 #3667 (Thermo Scientific) was transiently transfected into colon cancer cells according to the Dharmacon siRNA transfection protocol [46].

### Proliferation and apoptosis assays

For proliferation assays, cells were plated at 3x10^3^ cells per well in a 96-well plate. Five days later, cells were stained for 2 hours with 3-(4,5 Dimethylthiazol-2-yl)-2,5-diphenyltetrazoliumbromide (MTT) (Sigma). The OD at 570 nm was read on a ELx808^™^ Absorbance Microplate Reader (BioTek, Winooski) after the purple precipitates were dissolved in DMSO [47].

Apoptosis was detected using a DNA fragmentation ELISA assay kit (Roche) according to manufacturer’s protocol. Briefly, cells were seeded in 96-well plates and subjected to GFDS. Cells were stained with MTT to determine cell numbers or lysed for ELISA assays to determine apoptosis. Relative apoptosis was determined by dividing ELISA values by MTT values for each sample [47].

### Western blot analysis

Cells were lysed in TNESV buffer containing 50 mM Tris, 150 mM NaCl, 0.5% NP-40, 1 mM EDTA and 10% SDS, 5 mM Na3VO4, 50 mM NaF and 10 μg/ μL of β- glacerophosphate and 1 mM PMSF. Protein was separated by a 7.5% SDS-PAGE and transferred to a nitrocellulose membrane. The membrane was blocked using 5% milk in TBST at room temperature for an hour. After blocking, the membrane was incubated with a primary antibody followed by incubation with a secondary antibody. After washing, enhanced chemiluminescence detection was used to visualize the proteins of interest [45].

### Quantitative real time PCR

RNA was isolated from cells using the Roche high pure RNA isolation kit, according to the manufacturer’s instructions. Q-PCR analysis of IRS-1 mRNA was performed using Taqman Gene Expression Master Mix # 4369016 and the IRS-1 primer #HS00178563_M1 (Applied Biosystems) [48].

### *In vivo* xenograft studies

Animal experiments were approved by the University of Nebraska Medical Center (UNMC) Institutional Animal Care and Use Committee. FET control and DNRII cells (5 x 10^6^) were injected into the flank of 4–6 week old male BALB/c nude mice (Harlan Laboratories) that were randomized (n = 10). Tumors were measured every other day for 28 days. Tumor volumes (V) were calculated using the formula V = W^2^ × L × 0.5, where W represents the largest tumor diameter and L represents the next largest tumor diameter [32,47,49]. Upon termination of the experiments, tumors were dissected, placed in 10% neutral buffered formalin for 48hrs, and processed and embedded in paraffin for TUNEL, Ki67 and IHC analysis.

### TUNEL, Ki67 and IHC staining

Sections of formalin-fixed paraffin embedded (FFPE) xenograft tumors were mounted on slides and examined for proliferating and apoptotic cells by Ki-67 staining and TUNEL assay, respectively, as described previously [47,49].

IHC staining was performed on paraffin sections using the Novolink^™^ Min Polymer Detection System (Leica) as described previously [48,49]. Briefly, slides were subjected to antigen retrieval using Novocastra Epitope Retrieval Solutions, pH6, followed by incubation with an anti-IRS-1 antibody (1:700, Abcam, ab52167) or anti- p-IRS-1/2Y612 (1:400, Santa Cruz, sc-17195-R) overnight at 4°C. Slides were developed with DAB after incubation with Novolink polymer. Finally, slides were counterstained with hematoxylin. For each sample, ten randomly chosen fields were captured at 20 × magnification and quantified with Imagescope Software (Aperio Technologies, Inc.).

### Statistical analysis

Statistical analyses were performed using Student’s t-test and two-way ANOVA as indicated. A *P* value less than 0.05 was considered significant.

## Supporting information

S1 Checklist NC3Rs ARRIVE guidelines checklist. (PDF)
